# Supplementary material for: Complex‐centric proteome profiling by SEC‐SWATH‐MS
Source: Mol Syst Biol. 2019 Jan 14;15(1):e8438. doi: 10.15252/msb.20188438 (PMC6346213; doi:10.15252/msb.20188438)
Supplement: Supplementary file 8 — Dataset EV7 [file MSB-15-e8438-s008.zip › feature_plots_string/Q16763.pdf]

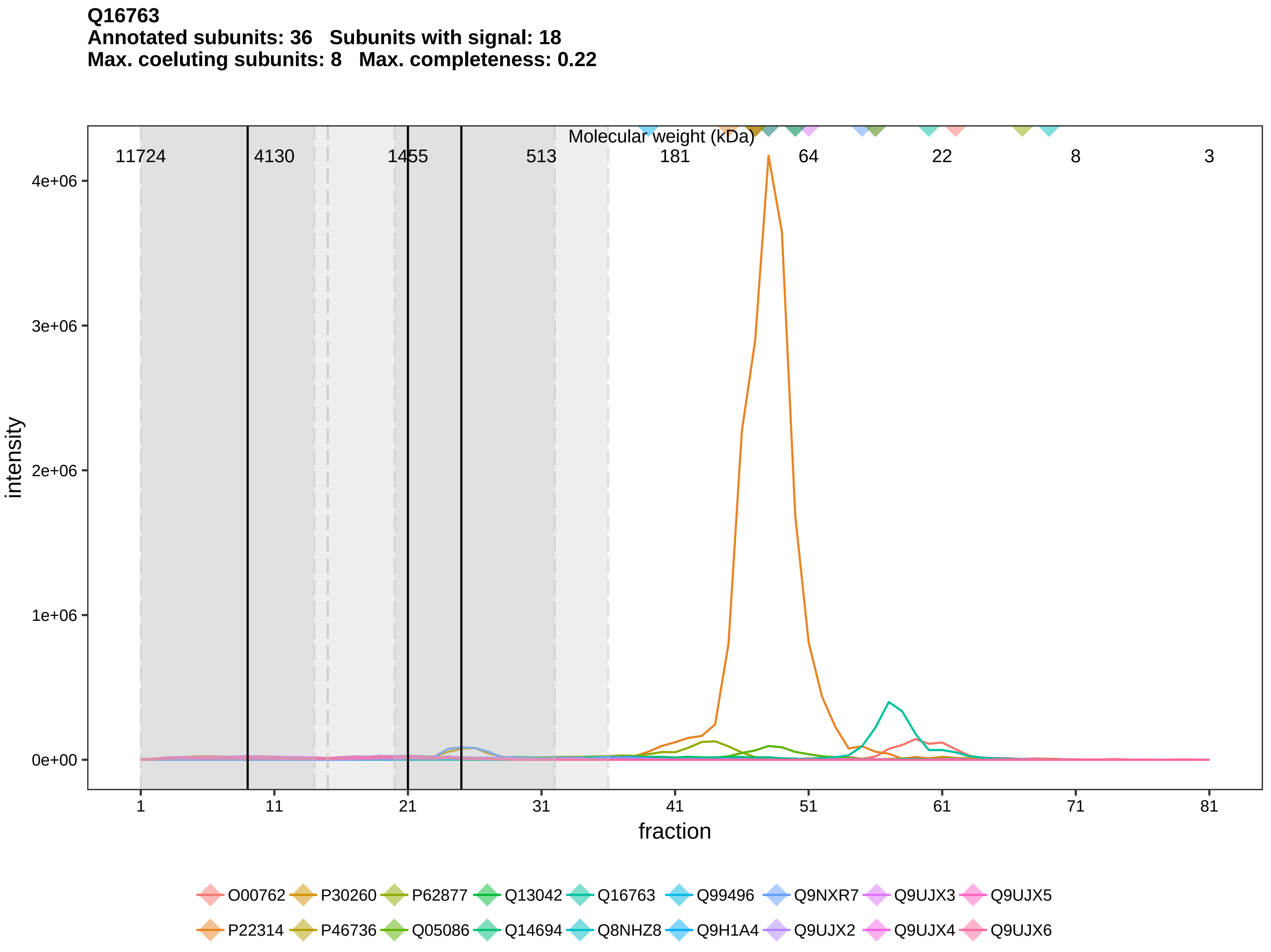

Q16763
Annotated subunits: 36 Subunits with signal: 18
Max. coeluting subunits: 8 Max. completeness: 0.22
Molecular weight (kDa)
11724
4130
1455
513
181
64
22
8
3
intensity
4e+06
3e+06
2e+06
1e+06
0e+00
fraction
1
11
21
31
41
51
61
71
81
O00762
P22314
P30260
P46736
P62877
Q05086
Q13042
Q14694
Q16763
Q8NHZ8
Q99496
Q9H1A4
Q9NXR7
Q9UJX2
Q9UJX3
Q9UJX4
Q9UJX5
Q9UJX6
